# Molecular features, biological behaviors and clinical implications of m^5^C RNA methylation modification regulators in gastrointestinal cancers

**DOI:** 10.1080/15384047.2023.2223382

**Published:** 2023-06-18

**Authors:** Mengyao Zhan, Huan Song, Dan Tian, Qin Wen, Xinling Shi, Yuting Wang, Xuhua Mao, Jianming Wang

**Affiliations:** a Department of Epidemiology, Center for Global Health, School of Public Health, Nanjing Medical University, Nanjing, China; b Department of Occupational Health, Suzhou Center for Disease Control and Prevention, Suzhou, China; c Department of Clinical Laboratory, Yixing People’s Hospital, Wuxi, China

**Keywords:** Epigenetics, transcriptome, methylation, 5-methylcytosine, prognostic biomarker, immunotherapy, gastrointestinal cancer

## Abstract

Epitranscriptome studies have shown that critical RNA modifications drive tumorigenicity; however, the role of 5-methylcytosine (m^5^C) RNA methylation remains poorly understood. We extracted 17 m^5^C regulators and clustered distinct m^5^C modification patterns by consensus clustering analysis. Gene set variation and single-sample gene set enrichment analysis were applied to quantify functional analysis and immune infiltration. The least absolute shrinkage and selection operator was employed to develop a prognostic risk score. Kaplan-Meier with log-rank test was used for survival analysis. Differential expression analysis was performed with the “limma” R package. Wilcoxon signed ranked test or Kruskal-Wallis test was used to compare groups. We observed that m^5^C RNA methylation was commonly upregulated in gastrointestinal cancer and related to prognosis. Clusters were identified for m^5^C patterns, with distinct immune infiltrations and functional pathways. The risk scores of m^5^C regulators were independent risk factors. Differentially expressed mRNAs (DEmRNAs) in m^5^C clusters were involved in cancer-related pathways. The methylation-based m^5^Cscore showed a significant effect on the prognosis. Patients with a lower m^5^Cscore exhibited more therapeutic efficiency on anti-CTLA4 therapy in liver cancer, while the combination of anti-CTLA4 therapy and pd1 was more efficient for patients with a lower m^5^Cscore in pancreatic cancer. We uncovered dysregulations of m^5^C-related regulators in gastrointestinal cancer and their associations with overall survival. Some immune cells were differently infiltrated in distinct m^5^C modification patterns, indicating their potential impacts on gastrointestinal cancer cell-immune. Moreover, an m^5^Cscore, derived from DEmRNAs in specific clusters, can serve as a classifier for immunotherapy.

## 1 Introduction

Over 170 distinct chemical modifications in RNA species have been identified and emerged as indispensable post-transcriptional regulators of gene expression.^1,2^ As far, multiple RNA modifications have been well identified in eukaryotes, especially for N6-methyladenosine (m^6^A), 5-methylcytosine (m^5^C), N1-methyladenosine (m^1^A) and pseudo-uridine (Ψ), etc.^3–6^ Among them, m^6^A modification has been regarded as the most prevalent and abundant mark since its discovery in the 1970s.^7^ The m^5^C RNA methylation, though in less abundance than m^6^A modification, has been extensively recognized in a variety of cellular RNAs, particularly in eukaryotic transfer RNAs (tRNAs), ribosomal RNAs (rRNAs), and messenger RNAs (mRNAs).^4,8,9^

Deposition of m^5^C modification is a dynamically reversible process, mediating by methyltransferases (“writers”), demethylases (“erasers”), and binding proteins (“readers”).^10^ In humans, m^5^C RNA methylation is generally catalyzed by NOP2/NSUN family (NSUN1–7) and DNA methyltransferase member (DNMT, DNMT3A, DNMT3B, and TRDMT1), with a residue specificity.^11^ Unlike well-documented “writers” for m^5^C RNA methylation, the existence of m^5^C “erasers” remains much to be elucidated. Recently, some documents have reported that the demethylation process predominantly relied on the ten-eleven translocator family (TET) and Alpha-Ketoglutarate-Dependent Dioxygenase AlkB Homolog 1 (ALKBH1).^12,13^ Two reader-binding proteins, Aly/REF export factor (ALYREF) and Y-box binding protein 1 (YBX1), can preferentially recognize the m^5^C-modified RNAs, thereby driving the biological process.^9,14^

Accumulating evidence has demonstrated that m^5^C RNA methylation is extensively implicated in physiological or pathological processes.^15^ In particular, these modifications regulate diverse RNA metabolism, modulating its structure, maturation, nuclear export, and protein synthesis.^14,16^ Reportedly, the m^5^C RNA methylations and regulators frequently altered in human cancers, indicating their potential functions as clinical biomarkers. For instance, NOP2 expression is overexpressed in breast cancer, gallbladder carcinoma, and lung cancer.^17–19^ The altered expression of NSUN2 has been widely linked to breast cancer, esophageal squamous cell carcinoma, or gastric cancer and correlated with the prognosis of patients.^14,20,21^ Mechanistically, m^5^C modification is frequently enriched in oncogenic pathways in bladder cancer.^14^ Intriguingly, the dynamic variation of m^5^C RNA methylation guarantees its rapid response to environmental stimulation, indicating its potential role in cancer therapy. Although m^5^C modifications have been widely described in human cancers, findings mainly focus on a limited number of m^5^C regulators in specific cancers.

Gastrointestinal cancer is a significant disease burden threatening human health. With an estimated 5.0 million new cases and 3.5 million deaths yearly, gastrointestinal cancer contributed 25.8% of global cancer incidence and 37.3% of cancer-related deaths in 2020.^22^ Survival of patients with gastrointestinal cancer tends to be poor, primarily ascribing to most diagnoses at an advanced stage. Thus, it is of great significance to identify noninvasive, sensitive, and specific biomarkers for the diagnosis and prognosis of gastrointestinal cancer. Epidemiological data have demonstrated that many gastrointestinal cancers are causally related to modifiable factors, including tobacco smoking, alcohol consumption, obesity, infection, and diet.^23–26^ Although gastrointestinal cancers share some common risk factors, their etiologies are mainly different. Recently, advances in high-throughput sequencing and bioinformatic techniques have accelerated the understanding of genetic alterations and epigenetic modifications. However, little progress has been made in the biological mechanism and clinical implications of m^5^C modification or its related regulators in malignant tumors, especially gastrointestinal cancers.

To investigate m^5^C-related regulators, clinical implications, and their correlations with the immune microenvironment, we integrated the transcriptome and genomic datasets of 1649 gastrointestinal cancer samples and 140 normal control samples based on The Cancer Genome Atlas (TCGA). We observed that m^5^C-related regulators were extensively altered in gastrointestinal cancer and linked to the overall survival of patients. Moreover, we constructed an m^5^Cscore to quantify the alterations for the patient for the first time, showing its potential function as a classifier for immunotherapy.

## 2 Results

### 2.1 Hallmarks of m^5^C RNA methylation regulators in gastrointestinal cancer

**Figure 1.**
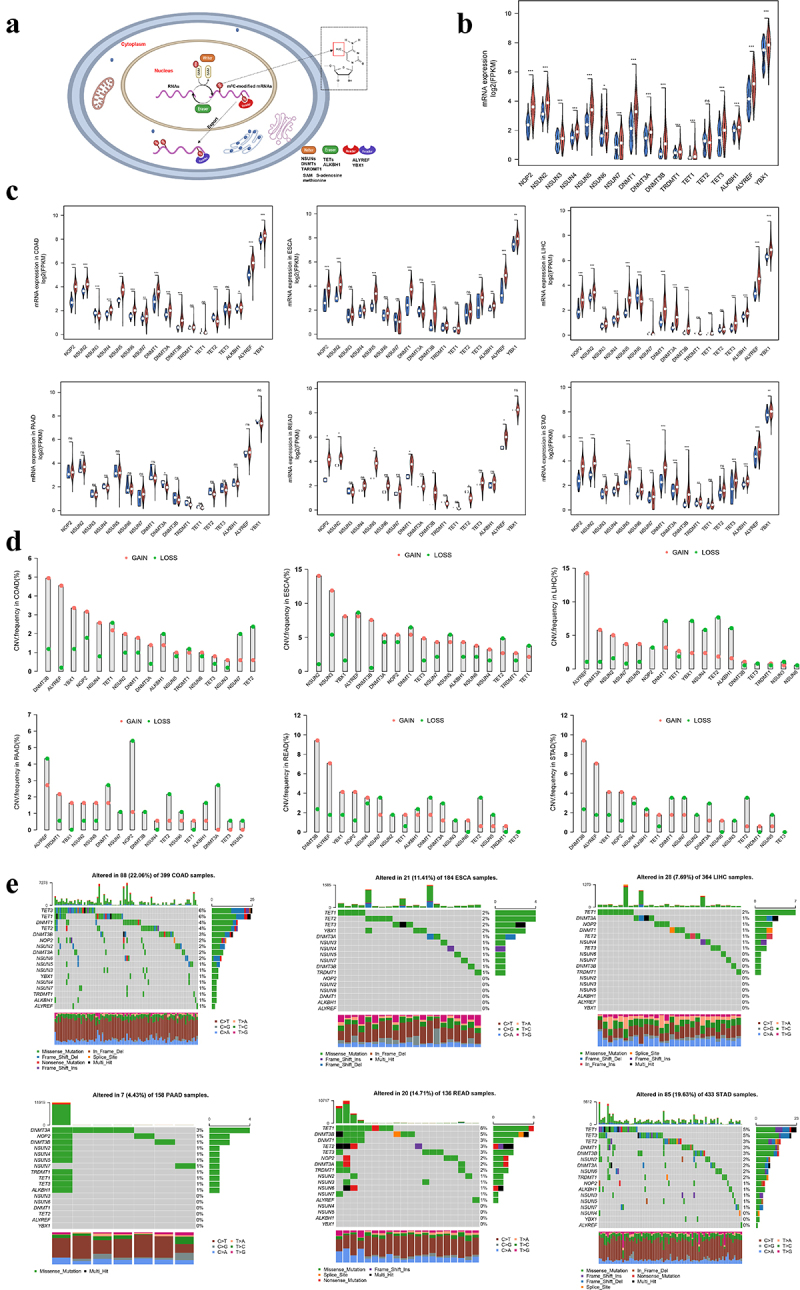
The integrated landscape of m^5^C regulators in gastrointestinal cancer.

A total of 17 well-defined m^5^C regulators were included in the analysis, including 11 “writers”, 4 “erasers,” and 2 “readers”. Concretely, the dynamic reversible process of m^5^C RNA methylation modification was schemed by methyltransferases, demethylases, and binding proteins in gastrointestinal cancer (Figure 1a). Considering the pivotal biological functions of m^5^C regulators, we systematically elaborated the landscape of m^5^C regulators in gastrointestinal cancer. Expression patterns analyses revealed that m^5^C regulators were commonly altered between 1649 patients with gastrointestinal cancer and 140 normal controls (Figure 1b). Specifically, most m^5^C-related “writers” and “readers” were overexpressed in each subtype of gastrointestinal tumors, except for pancreatic cancer (Figure 1c). Notably, only DNMT3A was downregulated in pancreatic cancer, while other regulators presented no differential expression.

(a) The schema of m^5^C RNA methylation dynamically meditated by m^5^C methyltransferases (Writers: NOP2, NSUN2, NSUN3, NSUN4, NSUN5, NSUN6, NSUN7, DNMT1, DNMT3A, DNMT3B and TRDMT1), demethylases (Erasers: TET1, TET2, TET3 and ALKBH1) and binding proteins (Readers: ALYREF, YBX1). (b) Vioplot visualizing the integrated expression of 17 m^5^C regulators both in tumor and normal samples in gastrointestinal cancer (Red: 1649 tumor samples; Blue: 140 normal samples). (c) Vioplot depicting the expression patterns of 17 m^5^C regulators in different types of gastrointestinal cancer. (d) The mutation frequency of m^5^C regulators in gastrointestinal cancer. (e) The copy number variation of regulators in gastrointestinal cancer. ns: Not significant; *: *P* <0.05; **: *P* <0.01; ***: *P* <0.001.

Then, we summarized the genetic variations of m^5^C regulators in gastrointestinal cancer. It was found that the copy number variation (CNV) alteration was prevalent in 17 m^5^C regulators (Figure 1d). Most regulators showed amplification in copy numbers, especially in colon cancer (COAD) and esophageal cancer (ESCA), while the CNV deletions were frequently found on TET2, ALKBH1, and DNMT1 (Figure 1d). The mutation frequency of m^5^C regulators was high in COAD (22.06%), followed by gastric cancer (STAD) (19.63%), rectal cancer (READ) (14.71%), ESCA (11.41%), liver cancer (LIHC) (7.69%), and pancreatic cancer (PAAD) (4.43%) (Figure 1e). Furthermore, the TET1 exhibited the highest mutation frequency in LIHC, READ, and STAD, while TET3 and DNMT3A were the most frequent in COAD and PAAD, respectively (Figure 1e). Together, these results demonstrated that the expression of m^5^C regulators was generally altered in gastrointestinal cancer.

### 2.2 Impacts of m^5^C RNA regulators on the prognosis of gastrointestinal cancer

Kaplan-Meier survival analysis revealed that dysregulated expression of m^5^C RNA regulators was associated with the overall survival of cases with gastrointestinal cancer (Supplemental Figure 1). Namely, decreased expression of 4 m^5^C “writes” (NOP2, NSUN5, NUSN6, DNMT3A) and 2 “erasers” (TET1 and TET3) was closely related to better overall survival, while overexpression of 4 “writes” (NSUN3 and NSUN4) and all ”readers” exhibited a better prognosis in COAD. For ESCA, downregulated NSUN4, NSUN6, DNMT1, and TET2 showed poor prognosis and downregulated DNMT3B and ALYREF presented favorable overall survival. Interestingly, all m^5^C RNA regulators were negatively related to the overall survival of patients with LIHC. For PAAD, patients with low expression of NSUN2, NSUN3, TET1, TET3, and YBX1 had an optimistic prognosis, while overexpression of NSUN6, DNMT3B, TET2, and ALKBH1 had a similar predictive effect. Overexpressed NOP2, NSUN2, NSUN3, NSUN4, and NSUN6 and lower NSUN2, DNMT3A, DNMT3B, and TET1 displayed a better prognosis of READ. Furthermore, overexpression of most m^5^C RNA regulators showed a distinctly superior survival advantage for cases with STAD, except for NSUN7, TET2, and ALKBH1.

**Figure 2.**
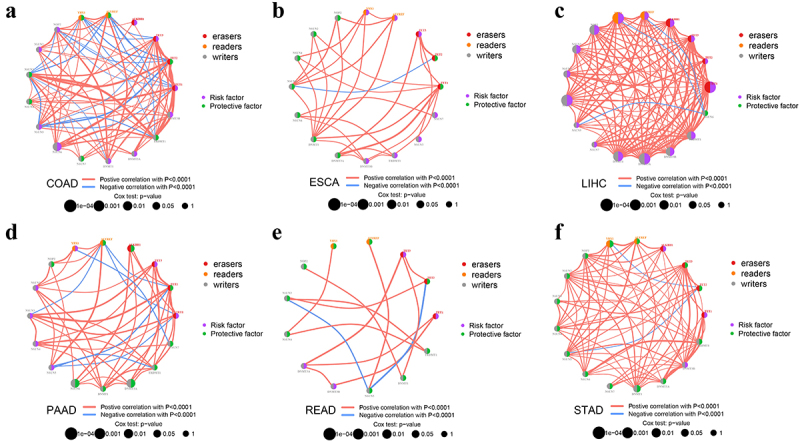
The correlations between 17 m^5^C regulators in gastrointestinal cancer.

Subsequently, we constructed a regulator network to depict the landscape of m^5^C regulator interactions and prognostic implications for patients with gastrointestinal cancer (Figure 2). We observed that these m^5^C regulators presented significant correlation in the internal categories of “writers”, “erasers”, and “readers”, and in external categories. There was a prevalent positive correlation among these 17 m^5^C regulators. The negative correlation between TET1 and NSUN5 existed in many types of gastrointestinal cancer, except for LIHC. For survival analysis, the expression of NSUN6 was identified as the risk factor for the prognosis of patients with COAD and almost every m^5^C regulator in LIHC. Reversely, DNMT3A, NSUN6, and ALKBH1 were protective factors for PAAD. Similar effects were found for YBX1 and DNMT1 in STAD. These findings indicated that m^5^C regulators’ cross-talk might be closely linked to the prognosis of patients with gastrointestinal cancer.

### 2.3 Significance of consensus clustering for m^5^C RNA regulators

**Figure 3.**
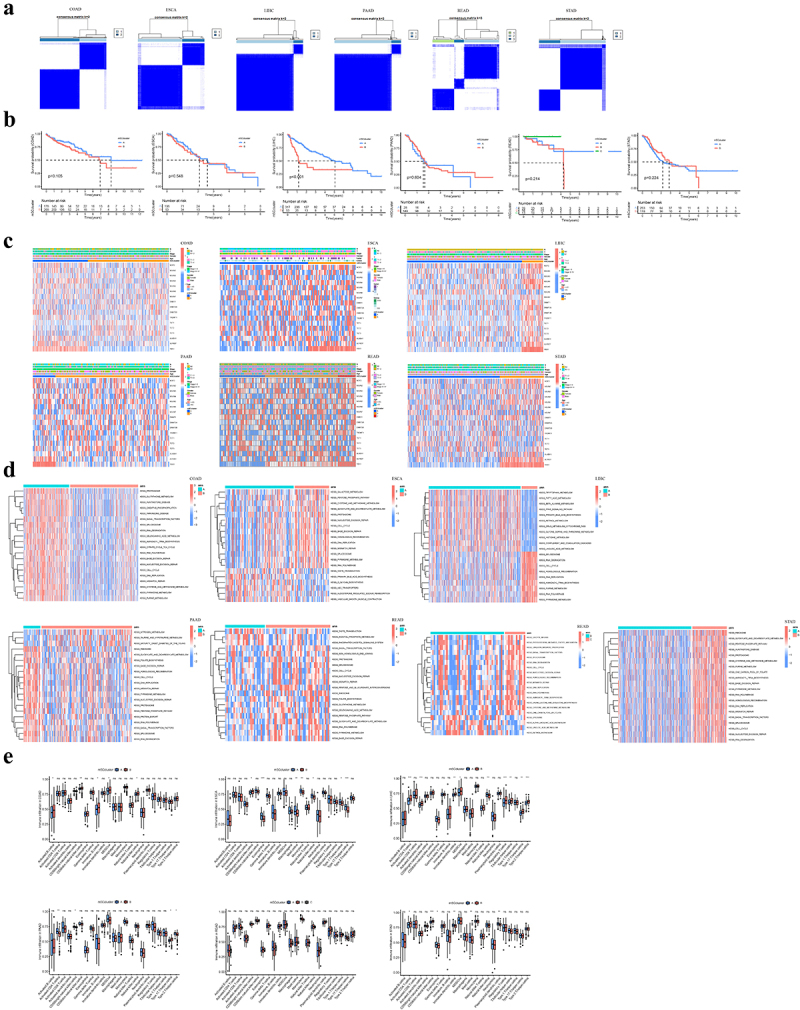
Distinct m^5^C RNA methylation patterns identified by Consensus clustering.

To further facilitate the potential biological behaviors and clinical implications of m^5^C regulators in gastrointestinal tumors, we conducted a consensus clustering analysis and eventually determined the optimal clustering for each subtype (terming as m^5^C clusters A-C) (Figure 3a). In terms of clinicopathological characteristics, we observed that m^5^C-related cluster A preferentially had higher overall survival in LIHC (Figure 3b). Besides, these m^5^C regulators were significantly differentially expressed in clusters of each type of gastrointestinal cancer, especially for YBX1 (Figure 3c).

(a) Consensus clustering identifying two or three subgroups of m^5^C RNA methylation patterns based on 17 m^5^C regulators. (b) Kaplan-Meier analysis of overall survival for distinct m^5^C RNA methylation clusters in patients with gastrointestinal cancer. (c) Heatmap showing the expression alterations of 17 m^5^C regulators in different m^5^C RNA methylation clusters. (d) Gene set variation analysis (GSVA) analysis displaying the biological pathways between m^5^C RNA methylation patterns. (e) Single-sample gene set enrichment analysis (ssGSEA) analysis revealing the infiltration of the immune cells between m^5^C RNA methylation patterns. ns: Not significant; *: *P* <0.05; **: *P* <0.01; ***: *P* <0.001.

As m^5^C modification patterns are frequently involved in the plethora of biological processes in human diseases, it is worthy of elucidating their underlying mechanisms in gastrointestinal cancer. For GSVA enrichment analysis, the results revealed that a series of molecular processes were enriched (Figure 3d). The metabolism, RNA degradation, cell cycle, and DNA replication were intriguingly identified as the universal difference among clusters of cancer subtypes. Given the frequent behaviors of m^5^C modification in cell immunologic processes,^27^ we further characterized the relative abundance of 23 immune cells in distinct clusters for each cancer subtype (Figure 3e). Among differentially enriched immune cells in COAD and ESCA clusters, the abundance of immune infiltration in cluster B was prevalently higher than in cluster A. Conversely, the immune infiltration of eosinophil and natural killer cells exhibited lower levels in cluster B than in cluster A in LIHC and activated CD4 T cells and CD56 natural killer cells in PAAD. However, no signs of immune infiltration were observed in READ. For STAD, the activated CD4 T cell and CD56dim natural killer cell were remarkedly enriched in m^5^C cluster B, while the other differences were prone in cluster A.

### 2.4 Construction of predictive models for m^5^C RNA regulators

**Figure 4.**
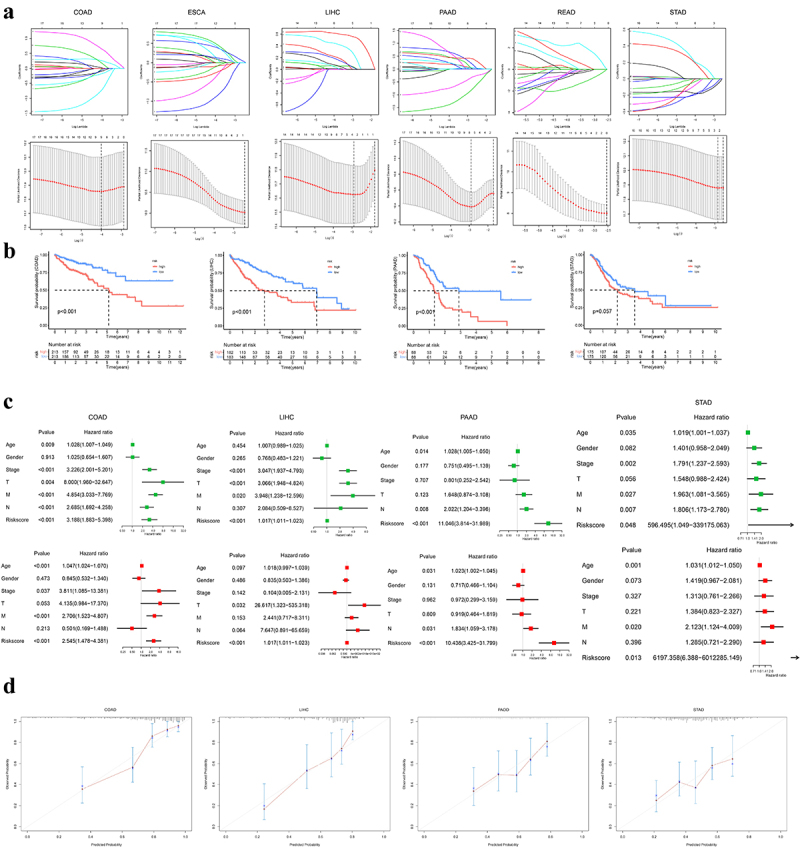
Construction of prognostic model based on m^5^C regulators.

To construct a prognostic model based on m^5^C regulators in patients with gastrointestinal cancer, we performed the least absolute shrinkage and selection operator (LASSO) Cox regression analysis (Figure 4a). Nine m^5^Cregulators were obtained as prognostic signatures of COAD, and the coefficients of these signatures were utilized to calculate the risk score $\;\left(Riskscore\approx NOP2\times 0.017-NSUN3\times 0.012+NSUN5\text{\textbackslash{}break}\times 0.258+NSUN6\times 0.641-NSUN7\times 0.094-TRDMT1\text{\textbackslash{}break}\times 0.719+ALKBH1\times 0.171-ALYREF\times 0.056-YBX1\text{\textbackslash{}break}\times 0.176\right)$. The risk score for LIHC was as follows: Riskscore=NSUN4×0.035+NSUN5×0.025+TET1\break×0.242×YBX1×0.551; and the formula for PAAD was as follows: Riskscore≈NSUN2×0.062+NSUN3×0.098+NSUN5\break×0.001−NSUN6×0.376−DNMT3A×0.884+DNMT3B\break×0.276+TET3×0.421+YBX1×0.047; and that for STAD was defined as $Riskscore\approx DNMT1\times \left(-0.072\right)\text{\textbackslash{}break}+YBX1\times \left(-0.044\right)$. However, no m^5^C-related prognostic signature was identified in ESCA and READ. Afterward, patients were subdivided into high- and low-risk subgroups based on the median risk score. As shown in Figure 4b, patients with high-risk scores were prone to inferior prognoses. Univariate and multivariate Cox regression models were employed to access the prognostic roles of age, sex, M stage, N stage, T stage, and clinical stage. Findings demonstrated that the m^5^C-related risk score was an independent risk factor for gastrointestinal cancer (Figure 4c). We also evaluated the discrimination and calibration of multivariate Cox regression models. The C-index was 0.783 (95% CI: 0.726–0.840) in COAD, 0.728 (95% CI: 0.667–0.789) in LIHC, 0.650 (95% CI: 0.589–0.711) in PAAD, and 0.652 (95% CI: 0.599–0.705) in STAD, respectively. Calibration plots presented good consistency between actual and predicted events (Figure 4d). These results suggested that the m^5^C corresponding risk model could serve as a prognostic classifier for gastrointestinal cancer.

(a) The prognostic model generated by the least absolute shrinkage and selection operator (LASSO) regression algorithm. (b) Kaplan-Meier analysis of overall survival for distinct m^5^C RNA risk score in patients with gastrointestinal cancer. (c) Univariate and multivariate Cox regression identified the independent prognostic signatures. (d) Calibration curves for prognostic models.

### 2.5 m^5^C-related DEmRNA patterns in gastrointestinal cancer

Although consensus clustering algorithms based on m^5^C regulators divided patients into distinct clusters, the underlying genetic alterations across clusters remained intriguing. Herein, we further explored these variations in expression patterns and biological processes. We first determined the different expressed mRNAs (DEmRNAs). A total of 2930 DEmRNAs between two clusters were considered the m^5^C-related signatures in COAD, followed by 129 DEmRNAs in ESCA, 8377 DEmRNAs in LIHC, 119 DEmRNAs, 1 DEmRNAs in READ, and 2905 in STAD. Given the limited DEmRNAs, READ was not involved in the following analysis.

**Figure 5.**
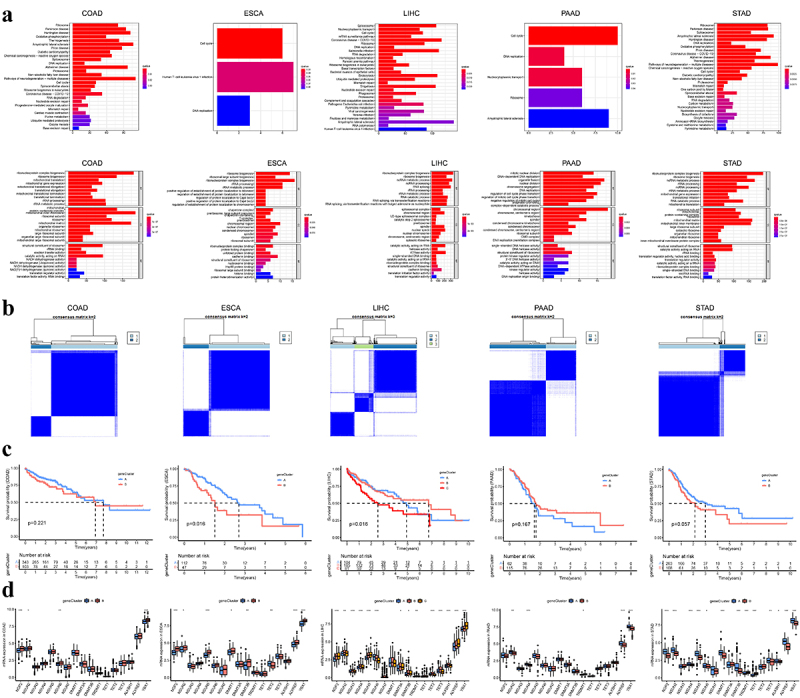
Identification of differential expression of m^5^C-related signatures and functional analysis.

Next, the ‘clusterProfiler’ R package was applied for GO annotation and KEGG enrichment pathway analysis; and the results demonstrated that some cancer-related pathways were enriched, including cell cycle, chemical carcinogenesis, and viral carcinogenesis (Figure 5a). We performed a consensus clustering analysis on the prognostic DEmRNAs to identify the genomic clusters (Figure 5b). The predictive differences in geneClusters were observed in patients with ESCA and LIHC, while those in other cancers were comparable (Figure 5c). To validate the m^5^C methylation modification patterns, we compared the abundance of m^5^C regulators. It was found that the expression patterns of m^5^C regulators significantly differed in geneClusters (Figure 5d).

(a) Functional analysis for m^5^C-related signatures using KEGG and GO enrichment analysis. (b) Consensus clustering identifying two or three subgroups of m^5^C geneClusters. (c) Kaplan-Meier analysis of overall survival for distinct m^5^C geneClusters in patients with gastrointestinal cancer. (d) Comparison of the expression of 17 m^5^C regulators between distinct m^5^C geneClusters. *: *P* <0.05; **: *P* <0.01; ***: *P* <0.001.

**Figure 6.**
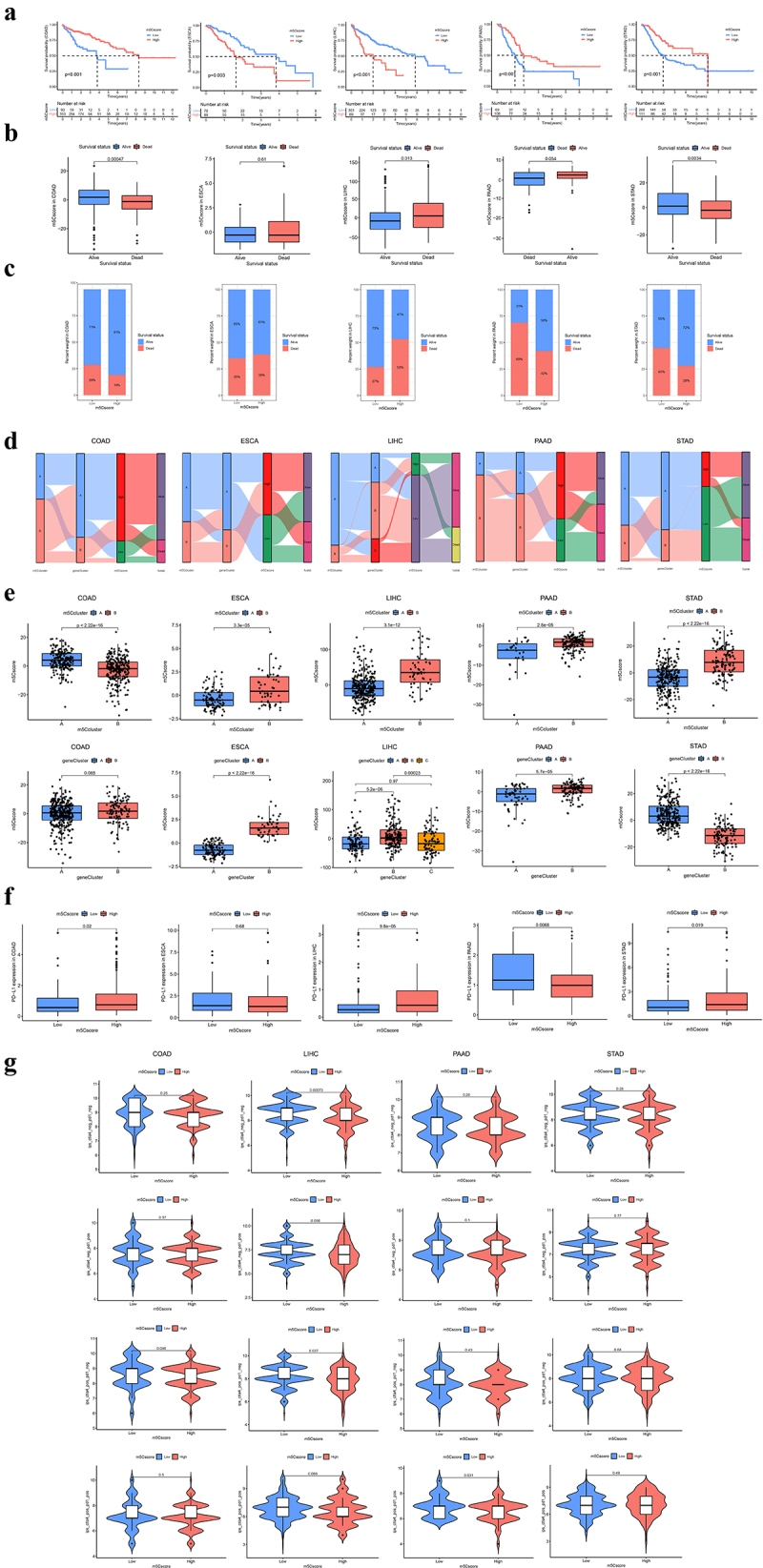
Generation of an m^5^Cscore and its clinical implications.

Based on the patient population, the observations demonstrated that m^5^C regulators exerted essential impacts on the biological process and prognosis of gastrointestinal cancer. Considering the individual heterogeneity, we generated an m^5^Cscore for particular m^5^C methylation based on DEmRNAs using the PCA analysis, followed by a classification of high- and low-m^5^Cscore. We found that a lower m^5^Cscore was associated with a worse prognosis in patients with COAD, PAAD, and STAD, whereas the observation conversed in ESCA and LIHC (Figure 6a). Consistently, a higher m^5^Cscore was observed in survivors compared to that of deaths in COAD and STAD, while deaths of LIHC tended to have a higher m^5^Cscore than that of survivors (Figure 6b,c). Specifically, the variations and association among the m^5^C cluster, geneCluster, m^5^Cscore, and survival status were illustrated by the alluvial diagram (Figure 6d). More importantly, the Wilcoxon test revealed a significant difference in m^5^C score between m^5^C clusters, and m^5^C clusters B tended to have higher scores, except for COAD (Figure 6e). Moreover, the geneCluster B presented with a higher m^5^C score, except for STAD.

(a) Kaplan-Meier analysis of overall survival for distinct m^5^Cscores in patients with gastrointestinal cancer. (b) Comparison of m^5^Cscore between different survival statuse. (c) Proportion of survival status in subgroups with high and low m^5^Cscores. (d) Alluvial diagram of m^5^C clusters in subgroups with different molecular and clinical subtypes. (e) Comparison of m^5^Cscore between distinct m^5^C clusters and geneClusters. (f) Relative expression of PD-L1 between subgroups with high and low m^5^Cscores. (g) Comparison of immunotherapeutic responses between subgroups with high and low m^5^Cscores.

### 2.6 m^5^C-related patterns in the role of anti-PD-L1 immunotherapy

Immunotherapy has emerged as a significant breakthrough in cancer treatment, especially for PD-L1 and PD−1 blockades. Herein, we explored whether m^5^C-related alterations could serve as a classifier for anti-PD-L1 immunotherapy. We observed that patients with a higher m^5^Cscore presented a higher abundance of PD-L1 in COAD, LIHC, and STAD; however, PAAD patients with a higher m^5^Cscore had a lower level of PD-L1 (Figure 6f). Additionally, patients with lower m^5^Cscore exhibited more therapeutic efficiency on anti-CTLA4 therapy in LIHC, while the combination of anti-CTLA4 therapy and pd1 was more efficient for PAAD patients with a lower m^5^C score (Figure 6g).

## 3 Discussion

“Epitranscriptome” studies have revealed the critical RNA modifications that drive tumorigenicity and development of cancers, such as m^6^A, m^5^C, m^1^A, and Ψ.^10^ Although m^5^C RNA methylation has been described in many RNAs, its expression patterns, biological behaviors, and clinical implications remain much to be elucidated. Here, we provided an integrated landscape of m^5^C RNA methylation regulators in gastrointestinal cancer based on public transcriptomic and genomic datasets. We found that the m^5^C regulators were widely altered in gastrointestinal cancers, and their originated risk scores correlated with the patients’ prognosis. We also identified distinct m^5^C modification patterns for each type of cancer and the dysregulation of immune cells among clusters. Moreover, the m^5^C score generated from DEmRNAs in clusters was associated with the expression of PD-L1 and immunotherapy.

Globally, gastrointestinal cancer remains a serious threat to public health, accounting for almost one-quarter of cancer incidence and one-third of cancer-related deaths in 2020.^22^ Although efforts have been made in diagnosis and therapy, the prognosis of the affected population remains dissatisfactory, primarily attributed to the delayed detection of cancers. Gastrointestinal cancer derives from environmental factors, genetic variations, and epigenetic changes. Accumulating evidence has demonstrated epigenetic alterations as diagnostic and prognostic biomarkers for gastrointestinal cancer, primarily owing to its frequency, stability, reversibility, and accessibility in body fluids and tissues. For instance, microRNA−7 (miR−7) has been identified as a diagnostic or prognostic biomarker in the colorectum, stomach, and liver cancers.^28–30^ The m^6^A modification of HDGF, meditated by METTL3, was an underlying prognostic and therapeutic target for gastric cancer.^31^

Previous studies have reported that some RNA methylation regulators were differentially expressed in gastric, colon, pancreatic, and hepatocellular carcinoma.^32–34^ These are mostly limited to a single tumor and lack cross-tissue comparisons. Our studies systematically disclosed that m^5^C regulators were widely dysregulated in gastrointestinal cancers, thereby serving as predictive biomarkers for affected patients. However, it should be noted that these expression patterns and prognostic implications showed much difference in distinct gastrointestinal cancer, presumably ascribing to their etiological differences. In particular, we also found that the genetic variations of m^5^C regulators varied in different gastrointestinal cancer. The TET1 mutation was most frequently in ESCA, LIHC, and READ, followed by TET3 in COAD and DNMT3A in PAAD. Moreover, the risk score derived from m^5^C regulators was identified as an independent risk factor for all gastrointestinal cancers. Collectively, our research provided an integrated landscape of m^5^C-related regulators in gastrointestinal cancer, as well as its clinical implications.

The tumor microenvironment (TME) includes fibroblasts, myofibroblasts, endothelial cells, and immune cells. As reported, cancer-associated fibroblasts (CAFs) and exosomes were extensively involved in the tumor microenvironment (TME), participating in the initiation, progression, dissemination, and treatment of human cancers.^35,36^ More interestingly, the past few decades have witnessed intensive efforts in therapeutic targets of immunotherapy for intervention in cancers.^37,38^ Many immunotherapy drugs have been approved, with numerous treatments in clinical or pre-clinical research. However, previous findings uncovered that only a subset of patients effectively responds to immunotherapies, challenging immunotherapy efficacy.^39^ Recently, the biomarker expression in cancers facility the classification of patient-specific immunotherapies. Zhang et al. reported that low m^6^A modification was related to an increased neoantigen load and enhanced response to anti-PD−1/L1 immunotherapy.^40^ Computational analysis revealed that long non-coding RNAs and immune checkpoints could predict the response to immunotherapy.^41^

Coincidentally, the functional analysis showed that several pathways were enriched in special m^5^C modification of gastrointestinal cancer, which may provide similarities for therapy. One study has reported that gene signatures could serve as prognostic predictors across different treatment regimens in breast cancer.^42^ However, our findings showed that the m^5^C regulators exert distinct effects on immunotherapy across different cancer types, indicating the heterogeneity of tumorigenesis of gastrointestinal carcinomas. Overall, our results uncovered the double-edged patterns of m^5^C modifications in specific gastrointestinal cancers, thereby serving as an inconsistent classifier for immunotherapies for different cancers.

It should be noted that several inevitable limitations were involved in this research. Namely, our findings originated from a publicly available database, and the pooled analysis based on more databases seems more interesting. More importantly, it is essential to validate our bioinformatics findings by experiment models.

## 4 Conclusion

Altogether, our results disclosed the extensive dysregulation of m^5^C RNA methylation regulators in gastrointestinal cancer and its correlation to overall survival. Several immune cells were found to be differently infiltrated in distinct m^5^C modification patterns, indicating their potential impacts on cell immunity in gastrointestinal cancer. Moreover, an m^5^Cscore, derived from DEmRNAs in different clusters was employed to quantify the level of m^5^C modification for patients with gastrointestinal cancer and thereby served as a classifier for immunotherapy.

## 5 Materials and methods

### 5.1 Data acquisition and pre-processing

The RNA-seq transcriptome data normalized by Fragments per Kilobase Million (FPKM) and corresponding clinical features of gastrointestinal cancer were accessed on July 17th, 2021, from the TCGA database (https://portal.gdc.cancer.gov/database). The gastrointestinal cancers we analyzed in this study included esophageal, gastric, hepatocellular, pancreatic, colon, and rectum adenocarcinoma. Specifically, there were 41 normal tissues and 473 tumor tissues of COAD, 11 normal tissues and 160 tumor tissues of ESCA, 50 normal tissues and 374 tumor tissues of LIHC, 4 normal tissues and 178 tumor tissues of PAAD, 2 normal tissues and 89 tumor tissues of READ, 32 normal tissues and 375 tumor tissues of STAD. Datasets of m^5^C-related regulators in each cancer were thoroughly queried. A total of 17 widely recognized regulators were simultaneously retrieved, containing 11 “writers” (NOP2, NSUN2, NSUN3, NSUN4, NUSN5, NUSN7, DNMT1, DNMT3A, DNMT3B, TRDMT1), 4 “erasers” (TET1, TET2, TET3, ALKBH1) and 2 “readers” CNV, were collected from the UCSC Xena data hubs (https://xena.ucsc.edu/). Tumor mutation load was defined as frameshift mutation, inflame mutation, splice site mutation, missense mutation, and nonsense mutation. The immunotherapy profiles were acquired from the Cancer Immunome Atlas (https://tcia.at/home).

### 5.2 Unsupervised consensus clustering for m^5^C RNA methylation regulators

To identify m^5^C regulators mediated subtypes in gastrointestinal cancer, we applied unsupervised consensus clustering to distinct tumor samples into subgroups based on the expression of m6A regulators using the ‘ConsensusClusterPlus’ R package. The parameters were set as pItem = 0.8 (resampling 80% of any sample), pFeature = 1 (resampling 100% of any sample), clustering algorithm = k-means, and distance = euclidean.

### 5.3 Gene set variation analysis and single-sample gene set enrichment analysis

Gene set variation analysis (GSVA) was introduced to investigate the biological process among m^5^C clusters using the ‘GSVA’ R package. Differential analysis was then performed to identify the variations in a biological process enriched in each cluster (adjust *P*-value <.05). The well-defined biological process was curated from the MSigDB database (http://www.gsea-msigdb.org/). The parameters for GSVA were as follows: minimum gene set size = 10 and maximum gene set size = 500. Single sample gene set enrichment analysis (ssGSEA) was performed to estimate the further infiltration of 23 immune cell types among clusters. The relative abundance of each immune cell type was calculated by an enrichment score and normalized into 0–1. A Gaussian fitting model assessed the bio-similarity of the infiltrating immune cells.

### 5.4 Generation of m^5^C-related prognostic signatures in gastrointestinal cancer

The least absolute shrinkage and selection operator (LASSO) Cox regression analysis generated m^5^C-related prognostic signatures in gastrointestinal cancers. In brief, the LASSO algorithm was utilized for signature selection and shrinkage using the ‘glmnet’ R package. The logarithmic-transformed expression levels of m^5^C RNA regulators were defined as an independent variable. The overall survival time and status of patients with gastrointestinal cancer were considered the response variables. The coefficients of each remained signature were derived from the LASSO regression analysis, and the risk score was generated using the formula of $riskscore\approx \overset{n}{\underset{k=1}{\sum }}\left(coefficient\;of\;mRNAk*expression\;of\;mRNAk\right)$. For subsequent analysis, patients were categorized into high-risk or low-subgroup based on the median value of scores.

### 5.5 Recognition of differentially expressed mRnas among distinct m^5^C RNA regulator clusters

The above consensus clustering algorithm was applied to categorize patients into different phenotypes. We further identified m^5^C RNA-related DEmRNAs among distinct clusters using the ‘limma’ R package (adjust *P*-value <.001). For function analysis of m^5^C RNA-related DEmRNAs in different clusters, the Gene Ontology (GO) and Kyoto Encyclopedia of Genes and Genomes (KEGG) analyses were carried out using the ‘clusterProfiler’ R package with *P* < .05 as a cutoff point.

### 5.6 Construction of m^5^C signature score

To quantify the altered patterns of gastrointestinal cancer, we used the principal component analysis (PCA) to generate the m^5^C signature score. Briefly, the DEmRNAs in distinct cancer clusters were recognized as candidates for survival analysis using a univariate Cox regression model. Among them, these DEmRNAs with prognostic significance were candidates for PCA analysis, and the principal components 1 and 2 were calculated for m^5^C signature score: $m^{5}c\;signature\;score=\sum \left(PC1k+PC2k\right)$.

### 5.7 Statistical analysis

We applied the R software version 3.6.1 (https://www.r-project.org/) to perform statistical analyses. Differential analyses of genes were performed using the ‘limma’ R package. The mutations of m^5^C regulators were illustrated by the ‘maftools’ R package. Wilcoxon signed ranked test or Kruskal-Wallis test was used to compare groups. Survival curves and differences were obtained by Kaplan-Meier survival analysis with a log-rank test. The hazard ratio (HR) was calculated using the Cox regression model and visualized by the ‘forestplot’ R package. We also applied the Harrell’s concordance index (C-index) and calibration curves to assess established models’ discrimination and calibration. A *P*-value less than 0.05 was considered statistically significant.

## Supplementary Material

Supplemental Material

## Data Availability

All data generated or analyzed during this study are included in this published article.
