# Supplementary material for: Molecular features, biological behaviors and clinical implications of m5C RNA methylation modification regulators in gastrointestinal cancers
Source: Cancer Biol Ther. 2023 Jun 18;24(1):2223382. doi: 10.1080/15384047.2023.2223382 (PMC10281471; doi:10.1080/15384047.2023.2223382)
Supplement: Supplemental Material [file KCBT_A_2223382_SM2478.docx]

**Supplementary Material**

**
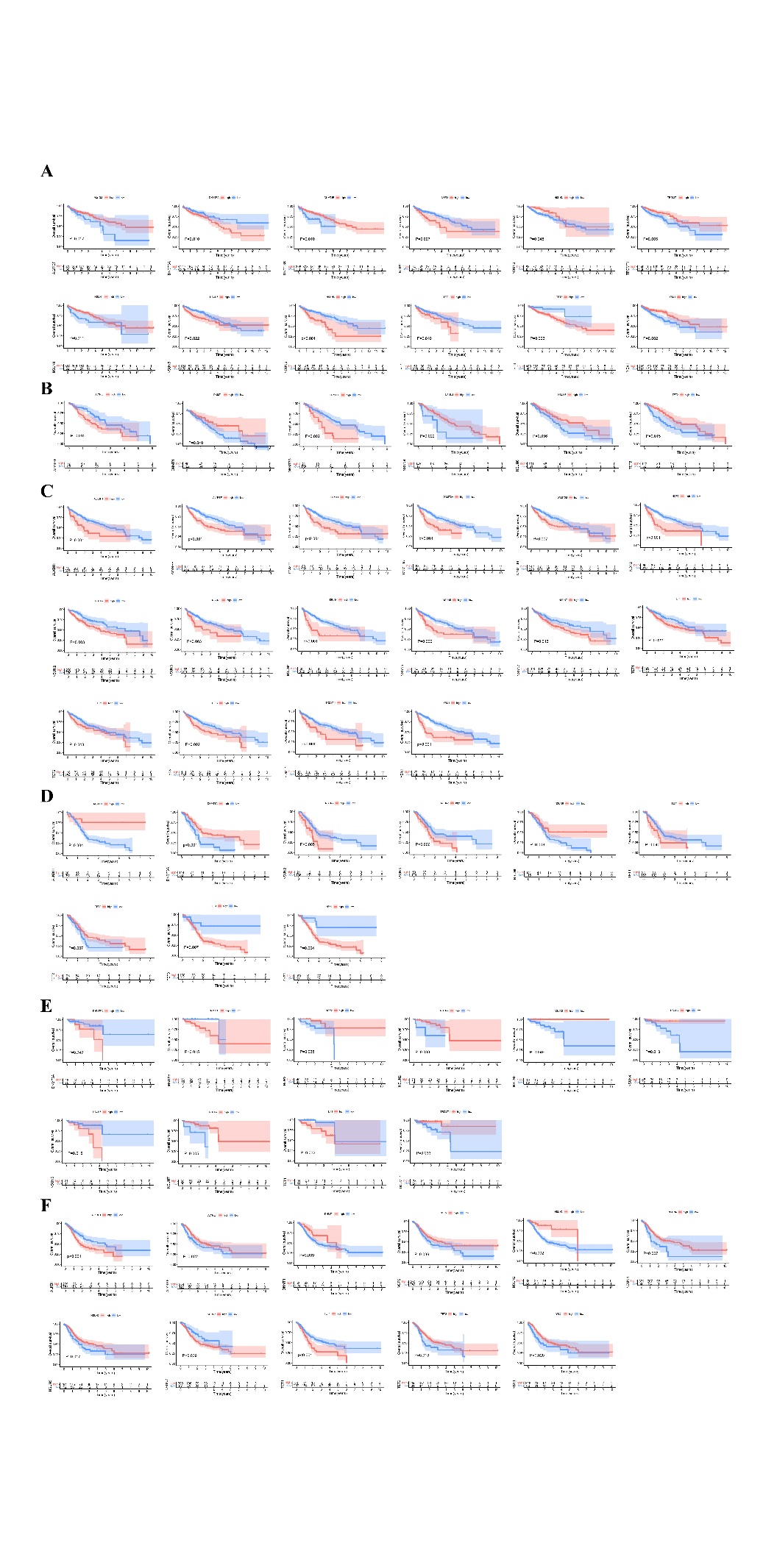
**

**Supplemental Figure 1.** Kaplan-Meier analysis of overall survival for m^5^C regulators in patients with gastrointestinal cancer.

(A) Kaplan-Meier analysis of overall survival for m^5^C regulators in patients with colon carcinoma (COAD). (B) Kaplan-Meier analysis of overall survival for m^5^C regulators in patients with esophageal carcinoma (ESCA). (C) Kaplan-Meier analysis of overall survival for m^5^C regulators in patients with hepatocellular carcinoma (LIHC). (D) Kaplan-Meier analysis of overall survival for m^5^C regulators in patients with pancreatic cancer (PAAD). (E) Kaplan-Meier analysis of overall survival for m^5^C regulators in patients with rectal carcinoma (READ). (F) Kaplan-Meier analysis of overall survival for m^5^C regulators in patients with gastric carcinoma (STAD).
